# Ranolazine for the symptomatic treatment of patients with chronic angina pectoris in Greece: a cost-utility study

**DOI:** 10.1186/s12913-015-1228-y

**Published:** 2015-12-18

**Authors:** Georgia Kourlaba, Charalambos Vlachopoulos, John Parissis, John Kanakakis, George Gourzoulidis, Nikos Maniadakis

**Affiliations:** The Stavros Niarchos Foundation-Collaborative Center for Clinical Epidemiology and Outcomes Research (CLEO), National and Kapodistrian University of Athens, School of Medicine, Athens, Greece; 1st Department of Cardiology, Athens University Hospital “Ippokrateio”, Athens, Greece; Heart Failure Unit, Department of Cardiology, Athens University Hospital “Attikon”, Athens, Greece; Department of Cardiology, “Alexandra” Hospital, Athens, Greece; Department of Health Services Organization & Management, National School of Public Health, Athens, Greece

## Abstract

**Background:**

To conduct an economic evaluation comparing ranolazine as add-on therapy to standard-of-care (SoC) with SoC alone in patients with stable angina who did not respond adequately to first line therapy, in Greece.

**Methods:**

A decision tree model was locally adapted in the Greek setting to evaluate the cost-utility of ranolazine during a 6-month period. The analysis was conducted from a third-party payer perspective. The clinical inputs were extracted from the published literature. The cost inputs considered in the model reflect drug acquisition, hospitalizations, vascular interventions and monitoring of patients. The resource utilization data were obtained from 3 local experts. All costs refer to the year 2014. Cost-effectiveness was assessed by means of the incremental cost per quality adjusted life year (QALY) gained with the ranolazine as add-on therapy relative to SoC alone (ICER). Probabilistic sensitivity analysis (PSA) was performed.

**Results:**

Ranolazine as add-on therapy was more costly compared to SoC alone, as the 6-month total cost per patient was €1170 and € 984, respectively. Patients received ranolazine plus SoC and SoC alone gained 0.3155 QALYs and 0.2752 QALYs, respectively. Ranolazine plus SoC resulted in an ICER equal to €4620 per QALY gained, well below the threshold of €34,000 per QALY gained. The PSA showed that the likelihood of ranolazine plus SoC being cost-effective at the threshold of €34,000 per QALY gained was 100 %.

**Conclusions:**

Τhe results suggest that ranolazine as add–on treatment may be a cost-effective alternative for the symptomatic treatment of patients with chronic stable angina in Greece.

**Electronic supplementary material:**

The online version of this article (doi:10.1186/s12913-015-1228-y) contains supplementary material, which is available to authorized users.

## Background

Chronic stable angina is one of the most common conditions experienced by patients with heart disease with significant detrimental effects on health related quality of life, including pain, poor general health status, psychological distress and restriction of activity [1, 2]. Moreover, there is evidence that patients with moderate or severe angina have more than a twofold higher risk for mortality compared to those with minimal or mild angina [3]. Apart from mortality and morbidity, chronic stable angina may impose great economic burden for payers, health care systems [4], and patients as this condition requires long-term pharmacological treatment, it leads to frequent hospital admissions, requiring in many cases expensive vascular interventions. Hospitalizations, both with and without revascularization procedures are the main cost drivers as they account for almost two thirds of the total health care expenditures related to chronic stable angina [4–6]. In addition to its direct medical costs, chronic angina has been found to lead to substantial productivity loss [7]. To be more precise, studies have shown that the total cost (both direct and indirect) of chronic angina is 2 to 3 times higher compared to the direct one alone [7].

In this context, it is clear that the effective management of chronic stable angina is important both for clinical and economic reasons. Effective treatment of chronic stable angina should aim at reducing or ideally abolishing symptoms, improve quality of life and prognosis [2]. Lifestyle changes, pharmacological therapy and revascularization procedures all have important roles in the management of stable angina [8–10]. The pharmacological management of stable angina includes, nitrates, b-blockers, calcium antagonists, statins, etc. [8–10]. Nonetheless, despite the aggressive use of conventional antianginal therapies, many patients experience persistent angina [11, 12]. For this reason, the need for additional antianginal agents with novel mechanisms of action arose. Several new drug have been tested for the management of chronic stable angina [13–16]. Ranolazine (Ranexa®) is one such antianginal agent [12]. Ranolazine is a drug that reduces angina symptoms, with a mechanism of action different from that of currently available pharmacological therapies [17–20]. It was approved on July 9, 2008 by the European Medicines Agency for use in patients with chronic angina, who continue to be symptomatic on b-blockers, and/or calcium antagonists [21].

The efficacy and safety of ranolazine as add-on treatment for stable angina has been evaluated in three clinical trials (MARISA [14], CARISA [17] and ERICA [22]) and one large trial of patients with non-ST elevation acute coronary syndrome (Merlin-TIMI 36 [23]). These clinical trials showed an improvement in exercise performance and a decrease in angina attacks.

Although, it is proven that ranolazine is an effective treatment option for the management of chronic stable angina, it may also impose a tangible cost to the health care system and payers. Greece is going through a significant economic crisis, which has resulted in major budget constraints on the national healthcare system. Under these circumstances, there is an increasing need for using therapeutic options which are not only clinically effective but also economically efficient, in order to maximize the value for money attained from the scarce resources invested in health care. In this context, the aim of the present study was to evaluate the cost-utility of ranolazine as add-on therapy to standard-of-care (SoC) compared to SoC alone in patients with stable angina who did not respond adequately to first line therapy with b-blockers and/or calcium channel antagonists in the health care setting of Greece.

## Methods

A decision tree model [24] was locally adapted to evaluate the cost-utility of ranolazine as add-on therapy to SoC, compared to SoC alone, during a 6-month period (time horizon) in patients with stable angina, who did not respond adequately to first line therapy with b-blockers and/or calcium channel antagonists in Greece. The analysis was conducted from a third-party payer perspective (National Organization for Healthcare Services Provision [EOPYY]). Because the time horizon did not exceed 1 year, no discounting was performed both to cost and health outcomes. The model led to calculation of incremental cost incurred and incremental quality adjusted life years (QALYs) gained using ranolazine as add-on treatment for stable angina. The cost-effectiveness of ranolazine was expressed as an incremental cost-effectiveness ratio (ICER) relative to SoC. As this study is an economic evaluation analysis and does not involve human subjects no ethics approval issues arise. Input data including human material or human data were derived from other published studies performed with the approval of an appropriate ethics committee [22, 23, 25–27].

### Model structure

**Fig. 1:**
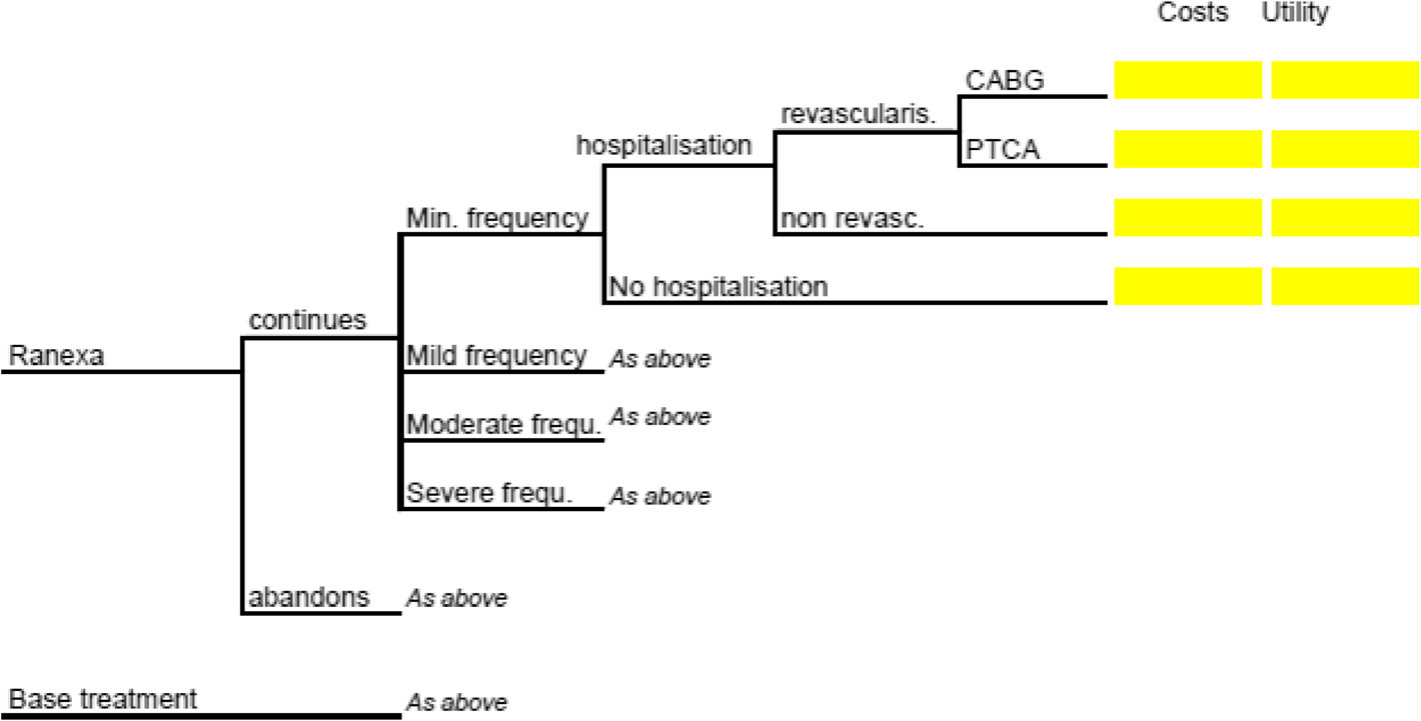
Model structure

The model used is a decision tree constructed on Microsoft Excel (Fig. 1), which has two main cohorts of patients representing both treatment alternatives. On the one hand, the ranolazine branch represents the treatment using ranolazine as an add-on therapy for the symptomatic treatment of angina pectoris. On the other hand, the “base treatment” branch represents the standard treatment in the same type of patients. The ranolazine branch is divided into two sub-branches, which shows the possibility of adherence or non-adherence by the patients to the treatment. Patients who do not adhere to the treatment with ranolazine are treated immediately with standard treatment, however for the purpose of the model and following the principle of intention to treat, these patients remain in the ranolazine branch. Whether or not the patient adheres to the treatment, four possible scenarios are considered, related to the frequency of angina that might be experienced at this stage of the model: minimal, mild, moderate or severe frequency. The transition probabilities depend on whether the patient has continued the treatment with ranolazine, whether the treatment has been stopped by the patient, or whether the patient belongs to the cohort of patients with standard treatment. Once the patient is in one of these potential angina frequency groups, hospitalization may or may not be required. When hospitalization is required, then an intervention might be needed or not. Most frequently required interventions at this stage are coronary artery bypass graft (CABG), or percutaneous coronary intervention (PCI). We did not consider mortality in this model related to stable angina pectoris, because, it has been shown in different studies that there are not significant differences in the mortality of patients under evaluation [23, 28].

### Clinical inputs

The clinical inputs considered in the model were: a) transition probabilities, including the probability of experiencing minimum, mild, moderate or severe angina frequency, the probability of being hospitalized, and the probability of being subjected to a revascularization procedure, and b) utility values related to non-hospitalization, hospitalization without revascularization, and hospitalization with revascularization. The above clinical inputs considered in the present analysis were obtained from relevant clinical and epidemiological trials [22, 23, 25–27]. It is worth noting that the same published studies were used in a previous publication of the model [24].

### Transition probabilities

As mentioned above, the transition probabilities considered in the present model are presented in detail elsewhere [24]. In brief, the probability of adherence to ranolazine treatment was estimated at 72 % [23] while the odds of experiencing at least a minimal frequency of angina (out of minimal, mild, moderate or severe) episode was estimated according to the treatment received [22] (Table 1). At this point it should be noticed that the frequency of angina classification depended on the self-administered questionnaire answers. The questionnaire used was the Seattle angina questionnaire [29]. It led to grouping of patients onto different categories according to their responses. To be more precise, patients with <0.5 episodes/week, 0.5-3 episodes/week, 3–5.5 episodes/week and > =5.5 episodes/week were classified to experience minimal, mild, moderate and severe angina, respectively.

**Table 1:** Clinical inputs considered in the model

	Value of the parameter	
	Ranolazine (%)	SoC (%)
Transition probabilities		
Adherence to treatment with ranolazine [23]	0.720	
Angina frequency [22]		
Minimum	0.040	0.000
Mild	0.605	0.071
Moderate	0.208	0.689
Severe	0.137	0.241
Hospitalization [25]		
With minimum angina frequency	0.200	0.200
With mild angina frequency	0.200	0.200
With moderate angina frequency	0.270	0.270
With severe angina frequency	0.300	0.300
Revascularization [26]		
Revascularization	0.500	0.500
Revascularization with CABG	0.280	0.280
Revascularization with PTCA	0.720	0.720
Utility values [27]		
No hospitalization, minimum angina frequency	0.810	0.840
No hospitalization, mild angina frequency	0.750	0.750
No hospitalization, moderate angina frequency	0.600	0.600
No hospitalization, severe angina frequency	0.390	0.390
Hospitalization without revascularization, minimum angina frequency	0.800	0.800
Hospitalization without revascularization, mild angina frequency	0.740	0.740
Hospitalization without revascularization, moderate angina frequency	0.590	0.590
Hospitalization without revascularization, severe angina frequency	0.380	0.380
Revascularization, minimum angina frequency	0.750	0.750
Revascularization, mild angina frequency	0.690	0.690
Revascularization, moderate angina frequency	0.540	0.540
Revascularization, severe angina frequency	0.330	0.330

*CABG:* coronary artery bypass graft, *PCI/STENT:* percutaneous transluminal angioplasty & stenting, *SoC:* standard of care

Moreover, the 6-month probability of hospitalization have been estimated at 20, 20, 27 and 30 % for minimal, mild, moderate or severe angina frequencies respectively, based on data obtained from Merlin-TIMI 36 clinical trial [25] (Table 1).

Of note, it has been found that the probability of hospitalization is independent of the type of treatment (ranolazine or SoC) but it depends on the severity of angina. When hospitalization is required, an intervention might be needed (or not) such as CABG or PCI/Stent [26] (Table 1). All these data were reviewed and validated by 3 local experts (C.V., J.P., J.K.) in order to increase the credibility and validity of the model inputs.

### Utility weights

QALYs were selected as the measure of effectiveness in present study. Utility weights are a measure of a patient’s preference of a particular health state and generally range from 0 (death) to 1 (perfect health). Utility values were obtained from the literature [27] (Table 1).

### Costing methodology

Since the analysis was conducted from the public third-party-payer perspective (EOPYY), only health care costs reimbursed by the payer were included in the model. The total reimbursement cost reflected and encapsulated all the possible healthcare resource consumption of patients during the horizon of analysis (6 months). The average resource utilization for a Greek patient suffering from stable angina was extracted from 3 the local experts using a questionnaire developed to serve the purpose of the present study. The questionnaire was developed to reflect the disease management in Greece, and the local experts participating in the present study confirmed its content validity. In this context, the cost of anti-anginal drug acquisition, hospitalizations, vascular interventions and monitoring tests including outpatient visits, laboratory tests and imaging diagnostic examinations were considered in the analysis. All costs reflect the year 2014 (€).

### Drug acquisition cost of anti-anginal drugs

Drug acquisition costs of ranolazine and SoC were calculated using the latest price bulletin issued by the Ministry of Health (26.11.2014) [30], as well as the corresponding reimbursement prices (Positive List for the reimbursement of medicines, FEK 3376/16.12.14). The reimbursement prices were reduced by the patient relevant patient co-payment (25 %) and relative rebates as they suggested by the corresponding legislation (Official Government Gazzete, FEK 64/16.1.2014). In particular, the rebate of 9 % given by manufacturers to get into the positive list was considered in the analysis. For ranolazine only, an additional rebate of 2 % was considered as it is alone in its cluster (Official Government Gazzete, FEK 64/16.1.2014). At this point, it should be pointed out that the volume-related rebate ranging from 2 to 12 % could have been considered in the analysis. Due to lack of volume –related data for all drugs, it was not taken into account in the base case analysis.

Standard care drug costs have been modelled using the overall proportions of patients using each therapeutic class and the mean daily dose for each drug as obtained from the 3 local experts and the relevant drug acquisition cost, calculated as mentioned above. For each therapeutic class, the active substances (INN) considered in the analysis reflected the most commonly prescribed drug locally, as obtained from the 3 local experts. Hence, a weighted cost was calculated for each therapeutic class based on the distribution of patients to different active substances. Based on those mentioned above, the total 6-month acquisition cost for SoC was calculated at 159.01€.

Regarding ranolazine, based on data provided by local experts it was considered that during the first two months 80 % of patients received 750 mg (375 mg twice daily) as a daily dose, while the remaining 20 % received 1000 mg (500 mg twice daily) as a daily dose and from the third month and onwards 50 % of patients received 750 mg (375 mg twice daily) as a daily dose, while 40 % of patients received 1000 mg (500 mg twice daily) as a daily dose and the remaining 10 % received 1500 mg (750 mg twice daily) per day. The total 2-month and 4-month acquisition cost for ranolazine was calculated at 89.27€ and 178.75€ respectively (Additional file 1: Table S1).

### Hospitalization costs

To estimate the total 6-month hospitalization cost (excluding hospitalizations related to vascular interventions), the reimbursement tariff per hospitalization obtained from the Diagnostic Related Groups (DRGs) tariffs’ list issued by the Greek Ministry of Health [31] was multiplied by the number of hospitalizations, as provided by the 3 local experts. The hospitalization cost at a 6-month period was found to range from €226.67 in patients experiencing minimal angina episodes to €2451 in patients experiencing severe angina episodes [Table 2].

**Table 2:** Other medical costs considered in the model

Medical cost for 6-month period				
Angina frequency	Hospitalization cost (€)^a^	Outpatient visits cost (€)^b^	Cost of laboratory tests (€)^c^	Cost of diagnostic tests (€)^c^
Minimal	226.67	7	7.93	40.72
Mild	453.33	9.17	9.41	64.55
Moderate	566.67	19.33	21.78	97.02
Severe	2450.83	31.33	40.29	123.79

^a^Official source: Based in local expert’s opinion and DRG such as K36X (€ 340) and K36M (€ 865) , DRG cost were obtained from FEK 9468/27-3-12

^b^Official source: This cost was obtained by combining of the resources (average of number of visits and % of patients), as they provided by the local expert’s opinion with the corresponding unit costs obtained from government gazette (FEK A’262/16-12-2011)

^c^Official source: The cost assigned was calculated based on resources used such as average of number of tests and % of patients). These resources were obtained from the local expert’s opinion and these resources were combined with the related unit costs obtained from official site of EOPYY

Regarding the cost of vascular interventions, the proportion of patients with stable chronic angina undergoing revascularization (i.e. PCI or CABG), as extracted from the literature [26] and validated by the 3 local experts, was combined with the reimbursed tariff per revascularization obtained from the relevant DRGs tariffs’ lists issued by the Greek Ministry of Health [31]. The cost per CABG and PCI/stent were found to be €5772 and €1798 respectively. To account for the effect of angina severity to the hospitalization cost, the model allowed the proportion of patients requiring hospitalization (with and without revascularization) and the hospitalization frequency to depend on angina severity.

### Routine monitoring costs

Routine monitoring costs include outpatient visits, laboratory tests (e.g. blood and biochemical tests) and diagnostic tests. The number of visits, laboratory tests and diagnostic tests (e.g. echo, MRI etc.) required as well as the proportion of patients undergoing each test were retrieved from the 3 local experts. The corresponding reimbursed unit costs were obtained from the Government Gazette (FEK A’262/16-12-2011) and from the official site of EOPYY respectively.

The 6-month cost for outpatient visits was found to be extremely low ranging from €7 in patients suffering from minimal angina episodes to €31.33 in those suffering from severe angina. With respect to laboratory and diagnostic tests, it was found that the 6-month laboratory test cost varies from €7.93 to €40.29 and the 6-month diagnostic tests cost from €40.72 to €123.79 [Table 2].

As with hospitalizations, the model allowed the proportion of patients undergoing to laboratory and diagnostic tests, as well as the number of tests and visits to depend on angina severity.

### Data analysis

The aforementioned data were used to get mean estimates of QALY, and total direct costs related to each comparator (ranolazine + SoC vs. SoC alone). The cost-effectiveness of ranolazine plus SoC over SoC alone was evaluated by calculating the incremental cost per QALY gained (ICER). For a treatment to be considered cost-effective a willingness-to-pay (WTP) threshold of €34,000 per QALY was used in the current analysis. This is based on the WHO guidelines stating that a treatment should be considered cost-effective if the ICER is up to 3 times the GDP per capita of that country and a treatment is considered highly cost effective at less than 1 times the GDP per capita [32]. The GDP per capita in Greece was estimated at €17,000 taken from the IMF estimation of GDP per capita using current prices [33].

The majority of input data used in the current model are subjected to variation. Therefore, in order to deal with uncertainty, a probabilistic sensitivity analysis (PSA) was performed using a second-order Monte Carlo simulation. In this analysis, a distribution was assigned around each parameter (i.e. costs, transition probabilities etc.) and the aforementioned economic and health outcomes associated with simultaneously selecting random values from those distributions were generated. Distributions were selected based on the nature of variables [34]. In particular, a gamma distribution was used to represent the uncertainty in costs, because costs are constrained on the interval zero to positive infinity and are often highly skewed. Binomial parameters (i.e. the probability of hospitalization) and utility values are constricted on the interval zero to one and hence they were varied according to beta distribution. For multinomial data such as the probability of experiencing a minimal, mild, moderate or severe angina episode, a Dirichlet distribution was used. Then, 5000 estimates of costs, LY, QALYs, and incremental cost per QALYs were obtained by applying the bootstrapping technique. The mean values of the bootstrapped estimates represent unbiased estimations of the parameters under investigation. The bootstrap percentile method was used to obtain the uncertainty appropriate intervals for each parameter [35]. A cost-effectiveness acceptability curve (CEAC) was plotted, showing the proportion of simulations that are considered cost-effective at different levels of WTP per QALY gained.

Moreover, a one-way sensitivity analysis (OWSA) was undertaken to test the robustness of the results, by varying individual parameters between low and high values, in order to ascertain the key drivers of cost-effectiveness. The upper and lower bounds of all parameters were set at ± 20 % (assumption) of the base case values. The parameters evaluated in this analysis were the cost of ranolazine, the cost of SoC, the cost of hospitalization with no revascularization, the cost of hospitalization with CABG, the cost of hospitalization with PCI/Stent, the probability of patients continuing on ranolazine therapy, the probability of hospitalization with minimal angina, the probabilities of hospitalization, and the utility values. Additionally, one scenario was also considered for the time horizon of the model, assuming that it was 1 year instead of 6 months used in the base case analysis.

All statistical calculations performed using Microsoft Excel 2010.

## Results

### Deterministic results

The analysis showed that, the total 6-month cost per patient was €1170 and € 984, for ranolazine plus SoC and SoC alone. In terms of health outcomes, the analysis revealed that ranolazine was more effective compared to SoC alone in terms of QALYs. Patients who received ranolazine plus SoC gained 0.3155 QALYs while patients who received SoC alone gained 0.2752 QALYs. Under the base case assumptions, incremental analysis showed that ranolazine plus SoC resulted in an incremental cost of €4620 per QALY gained, well below the predetermined WTP threshold of €34,000 per QALY gained [Table 3].

**Table 3:** Deterministic & probabilistic results for ranolazine plus SoC vs SoC alone

	Deterministic results			Probabilistic results		
Costs	Ranolazine	SoC	Incremental	Ranolazine	SoC	Incremental
				Mean (95 % CI)		
Total Costs (€)	1170	984	186	1170 (1123; 1213)	985 (933; 1036)	184 (154; 216)
Health outcomes						
QALYs	0.3155	0.2752	0.0403	0.3157 (0.3093; 0.3221)	0.2754 (0.2672; 0.2836)	0.0402 (0.0345; 0.0456)
Incremental analysis						
ICER per QALY (€)			4620			4904 (3526; 5962)

*ICER:* incremental cost effectiveness ratio, *QALY:* quality-adjusted life year, *SoC:* standard of care, *CI:* confidence interval

### One-way sensitivity analysis

The OWSA revealed that the results of the model were more sensitive to the utility for no hospitalization of mild angina severity as this parameter was found to have the greatest effect on ICER followed by the probability of patients continuing on ranolazine therapy and the utility for no hospitalization of moderate angina severity. It is worth noting that in all variations in the parameters which were considered in the sensitivity analysis the ranolazine remains a cost-effective treatment, since the ICER per QALY gained remains well below of the threshold of €34,000 per QALY gained.

Moreover, when the scenario of 1 year time horizon was considered in the analysis, ranolazine remained a cost-effective treatment resulting in an ICER equal to €6579 per QALY gained (data not presented in details) [Table 4].

**Table 4:** Results of one-way sensitivity analysis

Input parameter	Base case value	^a^Low value	ICER	^a^High value	ICER
Cost of ranolazine	€268	€214	€3511	€322	€5725
Cost of SoC	€159	€127	€4598	€191	€4643
Cost of hospitalization with no revas (mid, minimal, moderate)	€340	€272	€4692	€408	€4549
Cost of hospitalization with no revas (severe)	€865	€692	€4438	€1038	€4803
Cost of hospitalization with CABG	€5772	€4617	€4841	€6926	€4611
Cost of hospitalization with PCI/Stent	€1797	€1438	€4712	€2157	€4528
Probability of patients continuing on Ranolazine therapy	0.72	0.576	€7539	0.864	€2675
Probability of hospitalization with minimal angina	0.20	0.16	€4570	0.24	€4671
Probability of hospitalization with mild angina	0.20	0.16	€3858	0.24	€5393
Probability of hospitalization with moderate angina	0.27	0.21	€5572	0.31	€3684
Probability of hospitalization with severe angina	0.30	0.24	€5497	0.36	€3747
Utility for no hospitalization (minimal angina)	0.81	0.65	€4842	0.97	€4418
Utility for no hospitalization (mild angina)	0.75	0.60	€10,076	0.90	€2938
Utility for no hospitalization (moderate angina)	0.60	0.48	€3369	0.72	€7349
Utility for no hospitalization (severe angina)	0.39	0.31	€4391	0.47	€4875
Utility for hospitalization without revas (minimal angina)	0.80	0.64	€4647	0.96	€4594
Utility for hospitalization without revas (mild angina)	0.74	0.59	€4976	0.89	€4312
Utility for hospitalization without revas (moderate angina)	0.59	0.47	€4335	0.71	€4946
Utility for hospitalization without revas (severe angina)	0.38	0.30	€4572	0.46	€4670
Utility for hospitalization with revas (minimal angina )	0.75	0.60	€4645	0.90	€4596
Utility for hospitalization with revas (mild angina)	0.69	0.55	€4955	0.83	€4328
Utility for hospitalization with revas (moderate angina)	0.54	0.43	€4353	0.65	€4922
Utility for hospitalization with revas (severe angina)	0.33	0.26	€4576	0.40	€4666

*SoC:* standard of care, *CABG:* coronary artery bypass graft, *PCI/STENT:* percutaneous transluminal angioplasty& stenting, *Revas:* revascularization

^a^Low and high values currently based on ± 20 % variation from the base case

### Probabilistic sensitivity analysis

**Fig. 2:**
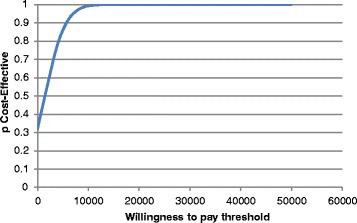
Cost-effectiveness acceptability curve ranolazine plus SoC vs SoC alone

The PSA confirms the deterministic results [Table 3]. In particular, the analysis showed that ranolazine was more cost-effective than SoC for all the simulations. The CEAC showed that the likelihood of ranolazine being cost-effective at a WTP threshold of 34,000€ was 100 %. Furthermore, in the alternative conservative WTP threshold of €17,000 the CEAC showed that the likelihood of ranolazine being cost-effective over SoC alone was 99.9 % [Fig. 2].

## Discussion

In the present study, an economic analysis was undertaken to compare, from a payer perspective, the ranolazine as add-on therapy to SoC relative with SoC alone during a 6-month period in patients with chronic stable angina in Greece. Ranolazine as add-on therapy to SoC was more expensive treatment regimen as it was found that the total 6-month treatment cost was higher by €186 compared to SoC alone. In terms of health outcomes, ranolazine was more effective as it reduces the severity and frequency of angina episodes in relation with SoC, resulting in higher QALYs gained. Hence, ranolazine was found to be a cost-effective treatment alternative, resulting in an ICER equal to €4620 per QALY gained well below the predetermined WTP threshold of €34,000 per QALY gained. Furthermore, the OWSA revealed that ranolazine as add-on therapy to SoC remains a well cost- effective treatment across all scenarios.

Our findings are in the line with those presented in the previously conducted economic analyses comparing ranolazine as an add-on therapy with SoC alone [24, 36, 37]. More specifically, a cost-utility study conducted in Spain [24] showed that ranolazine is a highly efficient add-on therapy for the symptomatic treatment of chronic angina pectoris with an ICER of €8455 per QALY. Moreover, a study which was conducted in the USA [36] showed that ranolazine as add-on to SoC is a cost-effective treatment in patients with weekly or daily angina with an ICER of $32,682 per QALY. Similar findings were obtained in a study conducted in Russia [37], which showed that the use of ranolazine in patients with > 3 attacks of angina pectoris per week was clinically and economically substantiated. Additionally, a retrospective study which performed in USA [38] showed that adding ranolazine to the treatment regimen of patients was associated with lower rates of revascularization and lower total costs of care than SoC (ranolazine:$13,961 vs nitrates :$18,166 and beta blockers/calcium channel blockers :$17,612).

The methodology adopted was based upon standard recommendations to conduct economic evaluation and extensive sensitivity and probabilistic analyses were conducted to fully explore uncertainty. However, potential limitations to this study should be considered. Firstly, in the present analysis it was assumed that the clinical data obtained from the published studies were applicable to the Greek health care setting. The use of this data may be questionable, however given the lack of local related data, this choice was the only source of relevant clinical data; one may argue that pivotal trials are almost universally used to build models for pricing and reimbursement decisions. Secondly, in the absence of local data, three local experts (cardiologists) with extensive clinical experience on the management of chronic stable angina were used to obtain local resource utilization and validate some of the assumptions considered in the model. This may raise concerns about the subjectivity of model inputs and leave space for challenging the study results. Nevertheless a series of sensitivity analyses indicated that our model and outcomes are valid, since the main results remained unchanged. Furthermore, the current analysis was conducted from the third party payer perspective and as such only direct costs were considered. However a more complete analysis from a broader (societal) perspective may also be worthwhile. True health care and patient direct and indirect costs are higher than those considered in the present analysis, and therefore the cost-utility of a new therapy may be more favorable from a societal perspective.

Finally, it should be noted that the results have to be considered in the strict Greek setting and on the basis of the present time resource and drug prices. If any of the underlying parameters change, so may the results and the conclusions of the analysis.

## Conclusions

The present study suggests that ranolazine as add-on treatment provides greater health benefits and is more costly. However, the additional cost per QALY gained is well below the predetermined WTP threshold of €34,000 per QALY gained. Therefore, the present study indicates that ranolazine as add-on treatment can be considered a cost-effective alternative for the symptomatic treatment of patients with chronic stable angina in Greece.

## Additional file

Additional file 1: Table S1. Drug acquisition cost considered in the model. (DOCX 79 kb)
