# Supplementary material for: Ranolazine for the symptomatic treatment of patients with chronic angina pectoris in Greece: a cost-utility study
Source: BMC Health Serv Res. 2015 Dec 18;15:566. doi: 10.1186/s12913-015-1228-y (PMC4683812; doi:10.1186/s12913-015-1228-y)
Supplement: Additional file 1: Table S1. — Drug acquisition cost considered in the model. (DOCX 79 kb) [file 12913_2015_1228_MOESM1_ESM.docx]

| Table S1: Drug acquisition cost considered in the model | | | | | | | | | |
| --- | --- | --- | --- | --- | --- | --- | --- | --- | --- |
| **Proportion of patients using each standard therapy, mean drug daily dose and relative drug acquisition cost** | | | | | | | | | |
| **Therapeutic Class** | **% of pts*** | | | **Cost inputs** | | | | | **6-month acquisition cost per therapeutic class (€)** |
| **Statins** | 88.33% | |  |  | |  | | |  |
| **Most commonly prescribed INN** |  | | **% of pts* per INN¶** | **Mean Daily Dose (mg)*** | **Cost per mg for each INN (€)**** | **Cost per day (€)** | **Cost for six months per INN (€)** | |  |
| Atorvastatin |  | | 58.52% | 27 | 0.0920 | 0.24 | 26.10 | | 48.43 |
| Simvastatin |  | | 14.07% | 30 | 0.0155 | 0.46 | 11.93 | |  |
| Rosuvastatin |  | | 20.74% | 13 | 0.0168 | 0.22 | 8.50 | |  |
| Pravastatin |  | | 3.33% | 40 | 0.0096 | 0.39 | 2.35 | |  |
| Ezetimibe |  | | 3.33% | 10 | 0.0979 | 0.98 | 5.95 | |  |
| **b-blockers** | 80% | |  |  | |  | | |  |
| Carvedilol |  | | 20.74% | 17 | 0.0075 | 0.13 | 4.74 | | 10.09 |
| Metoprolol |  | | 61.85% | 67 | 0.0004 | 0.02 | 2.69 | |  |
| Atenolol |  | | 10% | 50 | 0.0041 | 0.20 | 3.71 | |  |
| Bisoprolol |  | | 7.41% | 10 | 0.0109 | 0.11 | 1.47 | |  |
| **ACE Inhibitors** | 50% | |  |  |  |  |  | |  |
| Perindopril |  | | 28.33% | 8 | 0.0201 | 0.17 | 8.64 | | 21.53 |
| Ramipril |  | | 56.67% | 8 | 0.0367 | 0.31 | 31.61 | |  |
| Captopril |  | | 3.33% | 50 | 0.0018 | 0.09 | 0.55 | |  |
| Enalapril |  | | 11.67% | 15 | 0.0071 | 0.11 | 2.26 | |  |
| **Angiotensin II receptot blockers** | 23.33% | |  |  |  |  |  | |  |
| Valsartan |  | | 39.02% | 140 | 0.0010 | 0.14 | 10.10 | | 9.80 |
| Irbesartan |  | | 27.84% | 200 | 0.0010 | 0.20 | 10.30 | |  |
| Olmesartan |  | | 19.08% | 27 | 0.0131 | 0.35 | 12.62 | |  |
| Candesartan |  | | 13.33% | 16 | 0.0230 | 0.37 | 8.97 | |  |
| **Ca-blockers** | 40% | |  |  |  |  |  | |  |
| Amlodipine |  | | 59.21% | 7 | 0.0241 | 0.16 | 17.39 | | 11.02 |
| Nifedipine |  | | 13.51% | 33 | 0.0037 | 0.12 | 2.97 | |  |
| Diltiazem |  | | 23.95% | 180 | 0.0009 | 0.15 | 6.75 | |  |
| Veraramil |  | | 3.33% | 120 | 0.0006 | 0.07 | 0.44 | |  |
| **Long-acting nitrates** | 73.33% | |  |  |  |  |  | |  |
| Isosorbide Dinitrate |  | | 20% | 60 | 0.0017 | 0.10 | 3.66 | | 24.26 |
| Glyceryl trinitate (TTS) |  | | 18.89% | 8 | 0.0290 | 0.22 | 7.49 | |  |
| Isosorbide mononitrate |  | | 61.11% | 52 | 0.0038 | 0.20 | 21.93 | |  |
| **Antiplatelets** | 95% | |  |  |  |  |  | |  |
| Acetylsalicylic acid |  | | 77.54% | 100 | 0.0004 | 0.04 | 5.61 | | 30.68 |
| Clopidogrel |  | | 18.95% | 138 | 0.0053 | 0.74 | 25.42 | |  |
| Ticagrelor |  | | 2.63% | 90 | 0.0016 | 0.14 | 0.63 | |  |
| Prasugrel |  | | 0.88% | 10 | 0.0339 | 0.34 | 0.54 | |  |
| **Anticoagulants** | 20% | |  |  |  |  |  | |  |
| Acenocoumarol |  | | 60% | 2 | 0.0203 | 0.04 | 4.44 | | 3.19 |
| Dabigatran |  | | 3.67% | 110 | 0.0082 | 0.90 | 6.05 | |  |
| Rivaroxaban |  | | 2% | 10 | 0.1044 | 1.04 | 3.81 | |  |
| Apixaban |  | | 1% | 5 | 0.2091 | 1.05 | 1.91 | |  |
| **Drug acquisition cost of Ranolazine** | | | | | | | | | |
| **Ranolazine** | | **% of pts receiving  ranolazine *** | | **Daily Dose (mg)*** | **Cost per mg (€)**** | **Cost per day(€)** | | **Acquisition cost (€)** | |
| **Ranolazine for 4 months** | | 50% | | 750 | 0.0019 | 1.46 | | 89.20 € | |
|  |  | 40% | | 1000 | 0.0015 | 1.47 | | 71.64 | |
|  |  | 10% | | 1500 | 0.0010 | 1.47 | | 17.91 | |
|  | |  | |  |  |  | | **178.75** | |
| **Ranolazine for 2 months** | | 80% | | 750 | 0.0019 | 1.46 | | 71.36 | |
|  |  | 20% | | 1000 | 0.0015 | 1.47 | | 17.91 | |
|  |  |  | |  |  |  | | **89.27** | |
| Pts: Patients  *Official source : Based on local expert’s opinion  **Official source: latest price bulletin issued by the Ministry of Health (26.11.2014); corresponding reimbursement prices (Positive List for the reimbursement of medicines, Ministry of Health: Official Government Gazzete, FEK 3376/16.12.14) | | | | | | | | | |
